# High EpCAM expression is linked to proliferation and lauren classification in gastric cancer

**DOI:** 10.1186/1756-0500-6-253

**Published:** 2013-07-05

**Authors:** Feride Kroepil, Agnieszka Dulian, Daniel Vallböhmer, Helene Geddert, Andreas Krieg, Christian Vay, Stefan A Topp, Jan Schulte am Esch, Stephan E Baldus, Olivier Gires, Wolfram T Knoefel, Nikolas H Stoecklein

**Affiliations:** 1 Department of Surgery (A), Heinrich-Heine-University and University Hospital Duesseldorf, Moorenstrasse 5, 40225, Düsseldorf, Germany; 2 Department of Pathology, Heinrich-Heine-University and University Hospital Duesseldorf, Moorenstrasse 5, 40225, Düsseldorf, Germany; 3 Department of Otorhinolaryngology, Head and Neck Surgery, Grosshadern Medical Center, Ludwig-Maximilians-University of Munich, Munich, Germany; 4 Institute of Pathology St. Vincent’s Hospital, Karlsruhe, Germany

**Keywords:** Gastric cancer, EpCAM expression, Laurén-classification, Overall survival, Ki 67

## Abstract

**Background:**

The association of EpCAM expression with the progression of gastric cancer remains unclear. Here, we investigated the expression of EpCAM in gastric cancer subtypes and correlated the data to tumor cell proliferation and clinicopathologic factors.

**Methods:**

The intratumoral expression of EpCAM was assessed in 163 primary gastric cancers (61 diffuse-, 62 intestinal-, 32 mixed-type and 8 unclassified tumors) by immunohistochemistry, using the monoclonal antibody Ber-EP4. Intensity of staining was classified according the HercepTest-score using a standardized scoring system. Ki-67 was used to examine the proliferation in tumor tissue.

**Results:**

Strong EpCAM expression was observed in 77% of the tumors and in 85% of the corresponding lymph nodes. Of the primary tumors, 58% (n=74) presented a homogeneous intratumoral EpCAM expression while 42% were characterised by a heterogenous expression pattern. Tumors with high EpCAM expression at the invasive front were associated with significantly (p=0.03) higher proportion of lymph node metastases and lower median overall survival (p=0.001). Diffuse type tumors presented a significantly higher EpCAM expression at the invasion front compared with the tumor centre (p=0.036). Multivariate survival analysis identified high EpCAM expression at the invasive front as an independent prognostic factor.

We observed a significant (p=0.001) correlation between high EpCAM expression and higher tumor cell proliferation.

**Conclusion:**

High EpCAM expression associates with proliferation and progression of gastric cancer, especially in the diffuse type. Considering the discontenting results of the current adjuvant concepts for gastric cancer patients, EpCAM might be target in the adjuvant therapy of this malignant disease.

## Background

Despite improvements in preoperative staging, surgical techniques, and postoperative care, the outcome of patients with gastric cancer remains poor [1]. Although patients with stage I and II disease have 5 year survival rates of 83–99% (stage I) and 48–70% (stage II) after curative resection, the 5 year survival rate of patients with stage IV gastric cancer is only 25% [2]. As a consequence, multimodality therapy options have been established in order to treat patients with locally advanced gastric cancer [3]. However, effective prognostic/predictive markers identifying patients who might benefit from multimodal therapy are currently not available.

The epithelial cellular adhesion molecule (EpCAM) is a promising prognostic/predictive marker as well as an interesting molecular therapeutic target. EpCAM is a 37–42 kDa type I transmembrane glycoprotein with two epidermal growth factor like repeats in the external domain and a short intracellular domain containing of two α-actin binding sites for actin cytoskeleton linkage [4,5]. Recent studies demonstrated that EpCAM is a potent signal transducer that uses components of the Wnt pathway, with an active involvement of EpCAM in cell proliferation [6-8]. EpCAM is usually not expressed in healthy gastric mucosa but its de *novo* expression occurs frequently in gastric cancer [4]. In most tumor entities *de novo* and/or high EpCAM expression correlates with poor prognosis, conflicting data were published on the prognostic impact of EpCAM in gastric cancer [9,10].

In this immunohistochemical study of a large collection of gastric cancers we focussed on the relation between EpCAM expression and the Lauren classification system (morphology/differentiation) to further assess the potential role of EpCAM in different biological processes, including proliferation and differentiation, [6,9]. To additionally test the potential impact of EpCAM expression levels on proliferation, we stained consecutive sections of tumor tissues with the proliferation marker Ki-67 and correlated the staining data. Finally, we investigated the relation of the expression of EpCAM with clinicopathological factors and its impact on prognosis in gastric cancer.

## Results

### EpCAM expression in gastric normal mucosa and cancer

**Figure 1:**
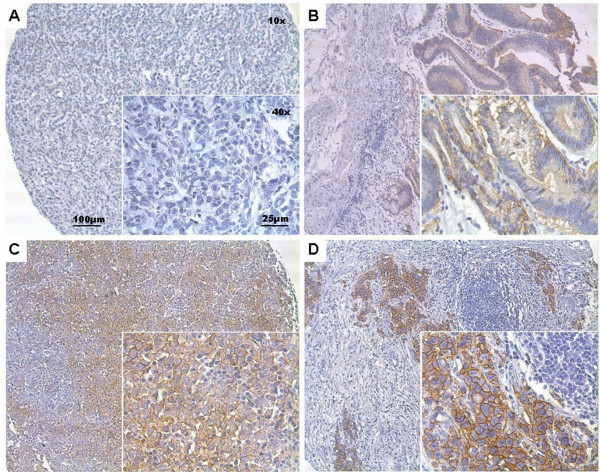
Examples of different staining levels of EpCAM in gastric cancer (A: EpCAM 0; B: EpCAM 1+; C: EpCAM 2+; D: EpCAM 3+).

As described previously [4], no expression of EpCAM was observed in normal gastric mucosa of all patients studied (100%, n=129). *De novo* expression of EpCAM could be observed in 77% (n=126) of gastric cancers. A strong (3+) EpCAM expression was found in 47 cases (29%), moderate (2+) expression in 27 cases (17%), and weak (1+) expression in 52 cases (32%) (Table 1). Absence of EpCAM was observed in 37 gastric cancer cases (23%). Representative examples of the abovementioned staining levels of EpCAM in gastric cancers are shown in Figure 1.

**Table 1:** Intratumoral EpCAM expression in all study patients

**EpCAM expression**					
	**0**	**1+**	**2+**	**3+**	**Σ**
patients	37 (23%)	52 (32%)	27 (17%)	47 (29%)	163
Primary tumor					
pT1	3 (14%)	10 (48%)	4 (19%)	4 (19%)	21
pT2	22 (26%)	25 (29%)	15 (17%)	24 (28%)	86
pT3	10 (21%)	13 (27%)	7 (15%)	18 (38%)	48
pT4	2 (25%)	4 (50%)	1 (13%)	1 (13%)	8
Lymph node status					
pN0	8 (20%)	13 (32%)	10 (24%)	10 (24%)	41
pN1	10 (19%)	19 (35%)	8 (15%)	17 (31%)	54
pN2	8 (29%)	10 (36%)	4 (14%)	6 (21%)	28
pN3	11 (28%)	10 (26%)	5 (13%)	13 (33%)	39
Tumor grade					
G1	1 (50%)	0 (0%)	1 (50%)	0 (0%)	2
G2	5 (14%)	11 (31%)	8 (22%)	12 (33%)	36
G3	30 (25%)	40 (33%)	18 (15%)	34 (28%)	122
G4	1 (33%)	1 (33%)	0 (0%)	1 (33%)	3
Laurén classification					
diffuse	20 (33%)	15 (25%)	9 (15%)	17 (28%)	61
intestinal	8 (13%)	21 (34%)	13 (21%)	20 (32%)	62
mixed	7 (22%)	12 (38%)	4 (13%)	9 (28%)	32
not classified	2 (25%)	4 (50%)	1 (13%)	1 (13%)	8

**Figure 2:**
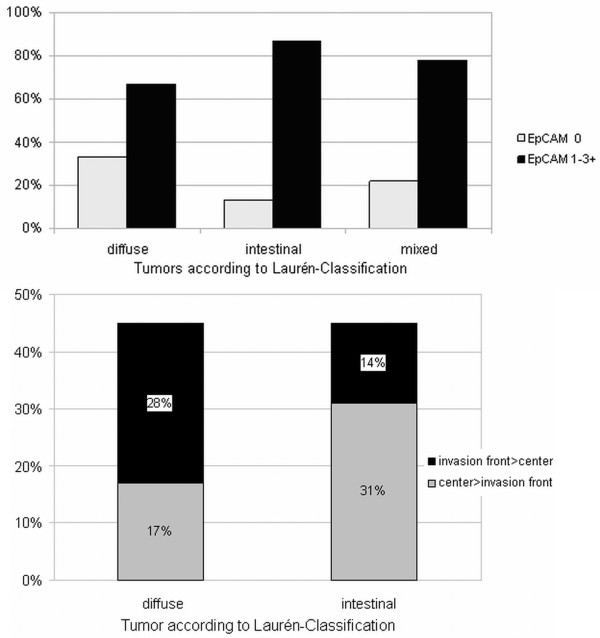
**EpCAM expression in different tumor compartments. A**: EpCAM staining in gastric cancer according to Lauren-classification **B**: Distribution (in %) of EpCAM staining pattern in diffuse and intestinal type gastric cancer. (Black = invasion front stronger than tumor center; light grey = tumor center stronger than invasion front); p=0.036.

In 42% of the study patients (n=54), we observed a differential EpCAM expression pattern with respect to the centre and the invasion front of the tumor. Tumors with higher EpCAM expression in the invasion front were associated with a significantly higher proportion of lymph node metastases (p=0.03; Table 2), whereas no significant correlations could be observed between EpCAM expression and pT-category, grading, or gender. Diffuse type gastric cancers exhibited a significantly weaker overall EpCAM expression than intestinal type cancers (p=0.008) or mixed type cancers (Figure 2A; Table 1). Under consideration of the Lauren-Classification we could observe that mixed type tumors were presented with the highest proportion of homogeneous EpCAM expression pattern (62.5% homogeneous, 37.5% heterogeneous). In contrast in diffuse and intestinal types of tumors 55% were presented with homogeneous and 45% heterogeneous EpCAM expression pattern. Focussing only the heterogenic expression pattern of 45%, we noticed that 28% of diffuse type of tumor was presented with higher EpCAM expression in the invasion front while the rest of 17% was presented with higher EpCAM expression in the tumor centre. In intestinal type tumors the EpCAM expression was high in the invasion front in 14% while 31% of the tumors exhibited high EpCAM expression in the tumor centre. Statistical analysis revealed that diffuse type gastric cancers were characterised by significantly (p=0.036) higher EpCAM expression in the invasion front (28%) than in the tumor centre compared to the intestinal type; (Figure 2B).

**Table 2:** **Correlation of intratumoral EpCAM expression pattern and pN**-**category**

	**pN-category**				
	**N0**		**N1-3**		**Significancy**
homogeous expression pattern	18	24%	56	76%	p = 0,065
heterogeous expression pattern	6	11%	47	89%	
tumor center > invasion front	5	61%	25	39%	p = 0,169
invasion front > tumor center	1	4%	22	96%	
invasion front > tumor center	1	4%	22	96%	p = 0,037
rest	23	22%	81	78%	

### Expression of EpCAM in lymph node metastases of gastric cancer

**Figure 3:**
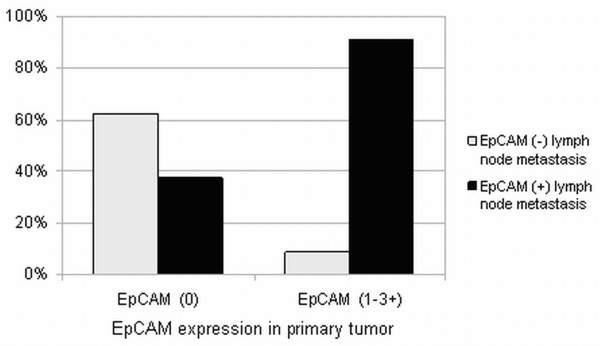
EpCAM staining in lymph node metastases and corresponding primary tumors (light grey=EpCAM-negative lymph node metastasis; black=EpCAM-positive lymph node metastasis).

Lymph node metastases of 88 cases were available for the analysis. Eighty-five percent (n=75) of lymph node metastases were positive for EpCAM. EpCAM-positivity of lymph node metastases was significantly correlated with EpCAM-positive primary tumors (p<0.001; Figure 3). Moreover, primary tumors with EpCAM-positive lymph node metastases displayed an advanced depth of tumor invasion (pT3/4) (p=0.046).

### KI-67 expression and its correlation to the expression of EpCAM

**Figure 4:**
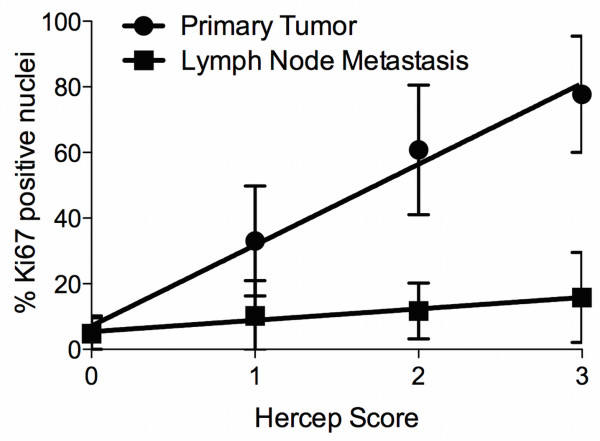
**Linear regression analysis of Ki-67 positive nuclei (% of tumor tissue) in correlation to the Hercep-Score of the EpCAM staining for primary tumor tissue and lymph node metastasis.** The slope of line for the primary tumor samples (n=163) was 24.64 [95% confidence interval (CI), 16.4 to 32.89] and the correlation coefficient (r^2^) 0.9881. For lymph node samples (n=88) the slope of the line was 3.466 [95% CI, 1.299 to 5.633] and the r^2^ 0.9595. The dots/squares illustrate the mean value and the error bars the standard error of the mean (SEM). The slope of the lines deviated both significantly from zero (primary tumor: p=0.006; lymph node metastasis: p=0.02).

**Figure 5:**
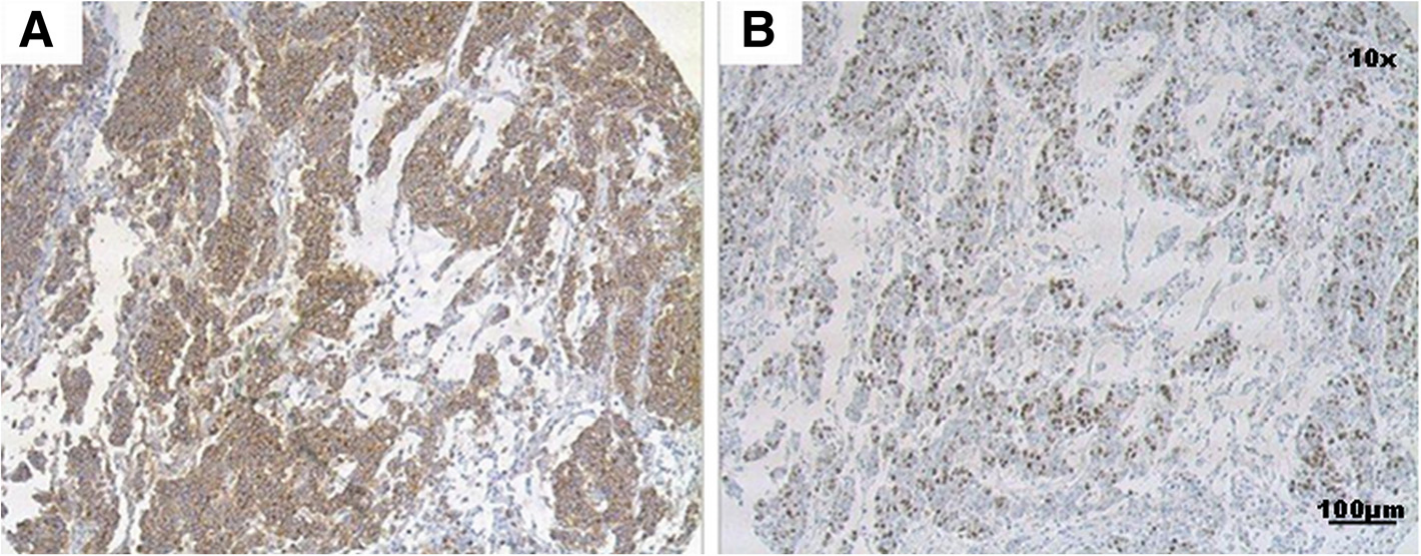
Example of A: EpCAM expression with corresponding B: Ki67 expression.

KI-67 positive nuclei were counted in 92% (n=147/160) of all gastric cancer samples with a median of 7% of KI-67-positive nuclei (range 0 to 60%). After classification into “high” proliferation (> 7% positive nuclei) and “low” proliferation (< 7% positive nuclei) according to the median proliferation rate, we observed a low proliferation rate in 57% and a high proliferation rate in 43% of the cases. Correlating EpCAM expression with KI-67-positive tumor cell nuclei using linear regression, we observed a significant positive correlation in primary tumor samples (p<0.006) as well as in lymph node metastases (p=0.02) (Figure 4). Figure 5 presents EpCAM expression in gastric cancer with corresponding Ki67 expression.

### Prognostic influence of EpCAM expression

For survival analysis, 135 patients could be included with a median observation time of 26 months (range 1–171 months). pT-, pN- category, and UICC-stage had a significant impact on overall survival (log rank, p<0.001), while gender, age, Laurén classification and grading showed no prognostic impact.

When comparing EpCAM negative tumors with EpCAM positive tumors, no significant correlation with overall survival was detected. The median cumulative survival was 20 months for EpCAM negative tumors and 24 months for patients with EpCAM positive tumors (log rank, p=0.765).

**Figure 6:**
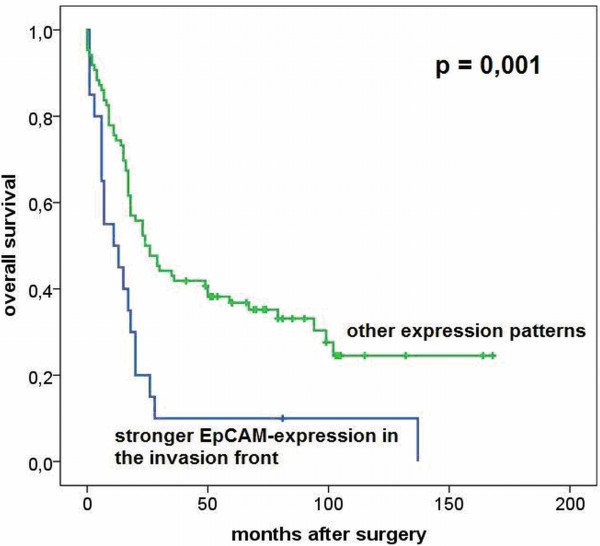
Survival analysis according to different EpCAM expression patterns in gastric cancer patients.

In contrast, a heterogeneous EpCAM expression pattern between the centre and invasion front of the tumor was associated with a significant negative impact on survival (log rank, p=0.04). The median cumulative survival time of patients with an increased EpCAM expression in the invasion front was the lowest with 11 months compared with 29 months of patients with a homogeneous EpCAM expression and 23 months in patients with EpCAM expression stronger within the tumor center (Figure 6).

When comparing the group of patients with a stronger EpCAM expression in the invasion front with all other patients, we found a significant negative prognostic impact (log rank, p=0.001). Performing multivariate analysis (cox-regression), we demonstrated that this was an independent prognostic factor for overall survival (Table 3).

**Table 3:** Univariate and multivariate survival analyses

		**Univariate analysis**					**Multivariate analysis**		
**Category**	**n**	**Deaths**	**% Alive**	**Mean survival(month)**	**95% CI**	**p-value**	**Relative risk**	**95% CI**	**p-value**
**Age**									
>70	71	46	35.2%	35	5.85-64.14	0.24			
≤ 70	64	46	35.2%	23	13.4-32.57				
**Gender**									
male	89	64	29.1	23	14.64-31.32	0.14			
female	46	28	39.1	51	3.1-98.83				
**pT-category**									
T1+2	98	61	62.2	56	16.21-95.78	0.000	2.0	1.2-3.2	0.003
T3+4	37	31	16.2	11	7.03-14.96				
**pN-category**									
N0	40	20	50	29.4	51.36-166.63	0.001	2.3	1.2-4.4	0.01
N1-3	95	72	24.2	2.6	14.09-25.0				
**Grading**									
G1+2	35	20	42.9	77	19.48-134.51	0.14			
G3+4	100	72	28	24	15.70-32.29				
**EpCAM Expr.**									
EpCAM IF+	20	19	5	11	0.0-24.14	0.001	2.0	1.2-3.5	0.008
Other exp. pattern	85	58	31.8	26	16.97-35.03				

## Discussion

This study investigated the expression pattern of EpCAM and its prognostic value in gastric cancer. We observed EpCAM expression in most of the gastric cancers, while it was absent in normal gastric epithelia. Interestingly, we observed differential EpCAM expression patterns with respect to the centre and invasion front of the tumor, which was identified as an independent prognostic factor. Furthermore, our study disclosed a significant correlation between high EpCAM expression and high tumor cell proliferation, suggesting that EpCAM promotes proliferation and progression of gastric cancer *in vivo*.

Although EpCAM belongs to the best studied cancer-associated antigens [11], its biological role in tumorigenesis is still not fully understood. While in some cancer types, including renal cell and thyroid cancer, EpCAM expression is associated with an improved survival, in other tumors high intratumoral EpCAM expression was identified as a poor prognostic factor [9,12-15]. Such controversial findings may have various biological reasons including the possibility of differing functions of EpCAM across organs and affected tissues. In this respect it is important to note that EpCAM is a potent signal transducer, which uses components of the Wnt pathway and which is causatively involved in the regulation of proliferation and cell cycle progression [5,7,16,17]. Regulatory functions of EpCAM however require regulated intramembrane proteolysis, and thus proteases, and interaction partners, which might represent limiting factors in some organs.

It is however puzzling to realise that even in the same entity, i.e. in gastric cancer, conflicting data on the prognostic impact of EpCAM were published [9,10]. For example, Songun et al. demonstrated in 300 gastric cancer patients that 93% were EpCAM positive and loss of expression identified aggressive tumors, especially in patients with stage I and II disease [18]. In contrast, *in*-*vitro and in*-*vivo* analyses by Du et al. showed that protein expression of EpCAM is higher in metastatic than in non-metastatic gastric cancers [10]. Similar findings were reported by Wenqi et al. in gastric cell lines and tumor tissues. The authors revealed that EpCAM is overexpressed in gastric cancer and down-regulation of EpCAM resulted in a decrease of cell proliferation and suppressed tumor formation [19]. One of the possible reasons to explain these different findings might be intratumoral heterogeneity of gastric carcinomas that was observed in our study. Obviosly, the different non-standardized immuno-histochemical methods and staining evaluations used in the different studies might also explain these discrepancies. Therefore we tried to use a relatively standardized scoring system the HercepTest (Dako), to classify the EpCAM expression. Using this system, we identified 77% of gastric carcinomas exhibiting *de novo* expression of EpCAM within the primary tumor, while normal gastric mucosa was devoid of EpCAM. These results are consistent with the findings reported by Songun et al. and Wenqi et al. However, while these authors were able to demonstrate a prognostic difference between EpCAM negative and positive tumors, we did not observe such a correlation. Interestingly, we found in 42% of our study patients differential EpCAM expression patterns when comparing the tumor centre with the invasion front. Tumors with higher EpCAM expression at the invasive front, exhibited a significantly higher proportion of lymph node metastases and a significantly decreased overall survival, which was of independent prognostic impact. Comparably, Gonsens et al. described a significant correlation between EpCAM staining at the invasive margin of rectal tumor specimens and tumor budding, tumor grade and an increased risk of local recurrence for the case of colorectal cancer [20]. Based on our findings, one might speculate about a differential intratumoral status of activation of EpCAM with a different expression pattern in invasion front and tumor center. On the other hand this difference seems not to be homogeneous in gastric cancer. This hypothesis has to be validated in further studies.

Moreover, we noticed in our study differential EpCAM expression patterns in the diverse types of gastric cancers according to the classification by Lauren. Similar findings have been shown by Joo et al. [21], who observed a correlation between intratumoral EpCAM over-expression and Lauren classification and histologic grading in gastric cancer patients.

As mentioned above, so far, the exact mechanisms of EpCAM contributing to the malignant potential of tumor cells are not entirely understood. *In vivo* experiments provided the first evidence of a direct link between EpCAM and cell cycle control. In fact, Chaves-Perez et al. recently showed that upon intratumoral EpCAM overexpression c-myc is rapidly upregulated [8]. EpCAM overexpression resulted in decreased growth factor requirement, enhanced metabolic activity and colony formation capacity. Importantly, expression of EpCAM anti-sense mRNA reversed these actions and led to a substantial decrease in proliferation and metabolism [22]. Furthermore, Maetzel et al. revealed that signalling by EpCAM requires regulated intramembrane proteolysis to release its intracellular domain EpICD. Released EpICD associates with components of the Wnt pathway, regulates gene transcription in the nucleus and, finally, cell proliferation and tumor formation [6]. Our study results are in agreement with these findings. In fact, we found that intratumoral EpCAM expression significantly correlates with Ki-67 expression, a nuclear proliferation associated antigen expressed in the growth and synthesis phase of the cell cycle [23]. These data suggest a higher proliferative activity in EpCAM positive cancer cells and an important role of EpCAM in cell cycle control as demonstrated *in vitro* by the tight regulation of cyclin D1 by EpCAM [8].

EpCAM expression might be also exploited for EpCAM-directed therapies. Just recently Ströhlein et al. performed an open-label, multicenter, phase I/II trial about the immunotherapy of peritoneal cancerosis with the antibody catumaxomab in gastric cancer [24]. In EpCAM-positive tumors catumaxomab, an antibody that specifically binds EpCAM-positive tumor cells and CD3+ T-lymphocytes, showed an acceptable safety profile and promising treatment efficiency, suggesting EpCAM to be an important target in the therapy of gastric cancer. Our results support these findings.

The present study provides some limitations. The relationship between intratumoral EpCAM expression and other known prognostic factors, such as lymphovascular invasion and serum CEA and CA19-9 would elevate the impact of this study. However this data were not available in the data base and could not be considered.

## Conclusion

We observed that high EpCAM expression is associated with increased proliferation and poor prognosis. Considering the discontenting results of the current (neo-) adjuvant concepts for gastric cancer patients, additional therapeutic EpCAM-targeting might be a beneficial concept for multimodality therapy of this malignant disease. Our findings are hypothesis generating and should be validated in larger clinical trials.

## Methods

### Tissue samples and clinical data

Paraffin-embedded tissue samples of 163 patients with gastric cancer were obtained from the institute of pathology for immunohistochemical analysis. The specimens were previously fixed in 10% formaldehyde, according to established methods [25]. There were 108 (65%) male and 57 (35%) female patients with a median age of 70 years (range 34–91).

All tissues were verified and graded in the pathology department. Tumor grading was performed according to World Health Organization (WHO) standards and according to Laurén-classification. The samples were randomly selected by experienced pathologists (S.E.B and H.G.) from the archives of the Department of Pathology of the University Hospital Duesseldorf based on availability of the follow up data. All patients underwent curative surgery at the University Hospital Duesseldorf between 1995 and 2006. Patients with neoadjuvant therapy were excluded from the cohort. Overall survival data were retrieved from a prospectively maintained clinical data-base of our hospital. Information about adjuvant therapy was not provided in this data base. Patients with metastasis, incomplete resection (R1) and/or incomplete data were not considered in the overall survival analysis. Tumor characteristics are summarised in Table 4.

**Table 4:** Tumor characteristics of the 163 study patients

		**n**	**%**
Primary tumor	pT1	21	13%
	pT2	86	53%
	pT3	48	29%
	pT4	8	5%
Lymph node status	pN0	41	25%
	pN1	54	33%
	pN2	28	17%
	pN3	39	24%
	pNx	1	1%
Metastases	M0	154	94%
	M1	9	6%
Tumor grade	G1	2	1%
	G2	36	22%
	G3	122	75%
	G4	3	2%
Laurén classification	diffuse	61	37%
	intestinal	62	38%
	mixed	32	20%
	not classified	8	5%

### Ethics statement

This study was approved by the Ethics Committee of the Medical Faculty of the Heinrich-Heine University Düsseldorf.

### Tissue microarray

Nine TMA-blocks were constructed, in which up to six tissue sample cylinders from 20 patients were arranged on one paraffin block. Two cylinders were chosen from the tumor centre, two from the invasion front, one sample of normal mucosa and one from a lymph node lymph node metastasis if available. For the tissue cylinders, a diameter of 1.0 mm was taken from representative areas of the donor blocks and transferred to the recipient TMA-blocks with interspaces of 2.5 mm between each tissue cylinder. In total, 835 (93%) tumor biopsies were analyzed.

### Immunochemistry

4 μm slides of each tissue microarray block were cut for immunhistochemical stainings. After deparaffinisation with xylol (Merck, 1.08685.2500; 3×10 minutes) and ethanol (99.5%, 2×5 minutes; 96%, 2×5 minutes; 80%, 2×5 minutes; 70% 1×5 minutes), epitopes were retrieved with Dako target retrieval solution at 95°C for 30 minutes and cooled down for another 20 minutes. Endogenous peroxidase was inactivated using 0.3% H_2_O_2_-PBS for 30 min at room temperature. Subsequently, sections were rinsed twice for five minutes in phosphate buffered saline (PBS, pH 7.4). Immunostaining was performed with antibodies directed against EpCAM (Ber-EP4, 2 μg/ml, Dako Cytomation, USA) and Ki-67 (mouse monoclonal, Dako Cytomation, USA, 1 μg/ml). Incubation with the primary antibodies was performed at room temperature for 30 min. The Vectastain ABC peroxidase kit was used following the manufacturer’s instructions (Vector Lab, USA) to visualize specific antibody binding. Isotype controls using IgG1 (MOPC-21, 2 μg/ml, Dako Cytomation, USA) were performed on serial sections of each sample. Diaminobenzidine (Liquid DAB, Dako Cytomation, USA) was used to stain the bound immuno-complex. All specimens were counterstained with haematoxylin (Sigma, Deisenhofen).

### Evaluation of immunhistochemical staining

Two independent researchers (AD, NHS) examined the sections. Differing results were discussed with a senior pathologist (SEB) and a consensus decision was made. Normal colonic mucosa was used as an internal control for staining efficiency. First, EpCAM staining intensity was classified according to the percentage of positive-stained cells into categories from 0 (no expression) to 3+ (strong expression) (Table 5). Then, tissue samples were classified according to the HercepTest™ (Dako) [26], a scoring system developed for the evaluation of p185HER-2-expression, which we applied for the evaluation of EpCAM expression [27,28]. Using this score, tumor specimens could be divided into four groups. The median of KI-67-positive tumor cell nuclei of all specimens was used to distinguish low (<7% positive nuclei) from high (>7% positive nuclei) proliferation.

**Table 5:** **HercepTest**-**Score for the quantification of EpCAM expression**

0	0-10% stained cells
1+	>10% weakly stained cells
2+	>10% moderately stained cells
3+	>10% strongly stained cells

### Statistical analysis

Statistical analysis was performed with the SPSS software (SPSS Standard version 17.0.0, SPSS Inc., Chicago, IL). P-values smaller than 0.05 (p<0.05) were considered statistically significant. Clinicopathological data were dichotomized. The *χ*^2^-test and the Fisher’s exact test were used to analyse the difference in the frequency of EpCAM-expression in relation to histopatholgical parameters. One-hundred-and-thirty-six patients were eligible for the analysis of overall survival. Survival curves were plotted according to Kaplan-Meier, univariate association was determined using the log-rank-test. Cox-Regression was performed to identify the independency of prognostic factors in a multivariate analysis.

## Competing interests

The authors disclose any financial and any non-financial competing interests.

## Authors’ contributions

FK participated in design and coordination and drafted the manuscript. HG and SEB collected and examined the probes and evaluated the immuno-assays. AD carried out and evaluated the immunoassays and helped to draft the manuscript. JSE, CV, AD and SAT performed the statistical analysis. NHS, WTK, OG, AK and DV participated in the design of the study, provided clinical data and helped to draft the manuscript. All authors read and approved the final manuscript.
